# Detection of *P. aeruginosa * harboring *bla *_CTX-M-2_, *bla *_GES-1_ and *bla *_GES-5, _*bla *_IMP-1_ and *bla *_SPM-1_ causing infections in Brazilian tertiary-care hospital

**DOI:** 10.1186/1471-2334-12-176

**Published:** 2012-08-03

**Authors:** Milena Polotto, Tiago Casella, Maria Gabriela de Lucca Oliveira, Fernando G Rúbio, Mauricio L Nogueira, Margarete TG de Almeida, Mara CL Nogueira

**Affiliations:** 1 Laboratório de Microbiologia. Departamento de Doenças Dermatológicas, Infecciosas e Parasitárias, Faculdade de Medicina de São José do Rio Preto, São José do Rio Preto, SP, Brazil; 2 Programa de Pós Graduação em Microbiologia, Universidade Estadual Paulista Júlio de Mesquita Filho, UNESP, Campus de São José do Rio Preto, Brazil; 3 Laboratório Central do Hospital de Base de São José do Rio Preto, São José do Rio Preto, SP, Brazil; 4 Laboratório de Pesquisa em Virologia. Departamento de Doenças Dermatológicas, Infecciosas e Parasitárias, Faculdade de Medicina de São José do Rio Preto, São José do Rio Preto, SP, Brazil

**Keywords:** *P. aeruginosa*, Nosocomial infection, ESBL, MBL, CTX-M-2, GES-1, GES-5, IMP-1, SPM-1

## Abstract

**Background:**

Nosocomial infections caused by *Pseudomonas aeruginosa* presenting resistance to beta-lactam drugs are one of the most challenging targets for antimicrobial therapy, leading to substantial increase in mortality rates in hospitals worldwide. In this context, *P. aeruginosa* harboring acquired mechanisms of resistance, such as production of metallo-beta-lactamase (MBLs) and extended-spectrum beta-lactamases (ESBLs) have the highest clinical impact. Hence, this study was designed to investigate the presence of genes codifying for MBLs and ESBLs among carbapenem resistant *P. aeruginosa* isolated in a Brazilian 720-bed teaching tertiary care hospital.

**Methods:**

Fifty-six carbapenem-resistant *P. aeruginosa* strains were evaluated for the presence of MBL and ESBL genes. Strains presenting MBL and/or ESBL genes were submitted to pulsed-field gel electrophoresis for genetic similarity evaluation.

**Results:**

Despite the carbapenem resistance, genes for MBLs (*bla*_SPM-1_ or *bla*_IMP-1_) were detected in only 26.7% of isolates. Genes encoding ESBLs were detected in 23.2% of isolates. The *bla*_CTX-M-2_ was the most prevalent ESBL gene (19.6%), followed by *bla*_GES-1_ and *bla*_GES-5_ detected in one isolate each. In all isolates presenting MBL phenotype by double-disc synergy test (DDST), the *bla*_SPM-1_ or *bla*_IMP-1_ genes were detected. In addition, *bla*_IMP-1_ was also detected in three isolates which did not display any MBL phenotype. These isolates also presented the *bla*_CTX-M-2_ gene. The co-existence of *bla*_CTX-M-2_ with *bla*_IMP-1_ is presently reported for the first time, as like as co-existence of *bla*_GES-1_ with *bla*_IMP-1_.

**Conclusions:**

In this study MBLs production was not the major mechanism of resistance to carbapenems, suggesting the occurrence of multidrug efflux pumps, reduction in porin channels and production of other beta-lactamases. The detection of *bla*_CTX-M-2,_*bla*_GES-1_ and *bla*_GES-5_ reflects the recent emergence of ESBLs among antimicrobial resistant *P. aeruginosa* and the extraordinary ability presented by this pathogen to acquire multiple resistance mechanisms. These findings raise the concern about the future of antimicrobial therapy and the capability of clinical laboratories to detect resistant strains, since simultaneous production of MBLs and ESBLs is known to promote further complexity in phenotypic detection. Occurrence of intra-hospital clonal dissemination enhances the necessity of better observance of infection control practices.

## Background

*Pseudomonas aeruginosa* is an opportunistic pathogen responsible for a large spectrum of invasive diseases in healthcare settings, including pneumonia, urinary tract infections and bacteremia [1]. For such infections, antimicrobial therapy may become a difficult task, because *Pseudomonas aeruginosa* is naturally resistant to many drugs, and presents a remarkable ability to acquire further resistance mechanisms to multiple classes of antimicrobial agents, even during the course of a treatment [2]. In this context, infections by *P. aeruginosa* presenting acquired resistance to beta-lactam drugs are considered one of the most challenging targets for antimicrobial therapy [3], being responsible for high rates of therapeutic failure, increase in mortality, morbidity, and in overall cost of treatment [4-6].

The Ambler’s class B beta-lactamases (metallo-beta-lactamases – MBLs) and class A extended-spectrum beta-lactamases (ESBLs) are acquired resistance determinants that present high clinical impact [7,8]. These enzymes, usually codified by genes associated with mobile genetic elements, are matter of major concern with regard to the future of antimicrobial chemotherapy because of its remarkable dissemination capability [9].

MBLs such as IMP, VIM, SPM, GIM and AIM represent the leading acquired mechanism of resistance to beta-lactams in *P. aeruginosa*. These enzymes hydrolyze the majority of beta-lactam drugs [10] and compromise clinical utility of carbapenems, the most potent agents for treating severe infections caused by multidrug resistant strains [11]. More recently, appearance of ESBLs such as TEM, SHV, CTX-M, BEL, PER, VEB, GES, PME and OXA-type beta-lactamases has become an emergent public health problem, since these enzymes confer resistance to at least all expanded spectrum cephalosporins and compromise efficiency of ceftazidime, an important antibiotic regimen for *P. aeruginosa*[12-14].

For the establishment of the appropriate antimicrobial therapy and for assessment and control of the spread of drug resistant *P. aeruginosa*, the molecular detection and surveillance of resistance genes is becoming increasingly important [15,16]. Despite of this, epidemiological data reporting the prevalence of MBL and ESBL producing *P. aeruginosa* is sparse, due to the inexistence of standardized methods for phenotypic detection of ESBL and MBL production [7,17] and the complexity for the implementation of PCR based methods in the routine of clinical laboratories [17]. In order to overcome this difficulty, a number of commercial rapid molecular tests are being developed that identify pathogens and the presence of genetic determinants of antimicrobial resistance [18,19]. The association of such new technologies with current classical methods may improve the ability of clinical laboratories to provide accurate and fast results that will impact on patient care.

Hence, this study was performed to investigate the carriage of genes codifying MBLs and ESBLs by *P. aeruginosa* isolated from patients admitted to a Brazilian 720-bed teaching tertiary care hospital. Despite the high rates of carbapenem resistance, genes for MBLs production were not observed in the majority of the isolates. Notably ESBLs codifying genes *bla*_CTX-M-2_, *bla*_GES-1_ and *bla*_GES-5_ were detected in several strains, and the coexistence of *bla*_CTX-M-2_ with *bla*_IMP-1_, and *bla*_GES-1_ with *bla*_IMP-1_ in *P. aeruginosa* was observed, and is presently reported for the first time. These findings underline the emergence of class A extended-spectrum beta-lactamases among *P. aeruginosa.*

## Methods

### Bacterial isolates

In this study were included a total of 56 *P. aeruginosa* isolates resistant to ceftazidime, imipenem and/or meropenem, nonrepetitively obtained from patients admitted to a teaching hospital in São Paulo State, Brazil, over a period between June to December 2009. Isolates were obtained from specimens originated from urinary tract, respiratory tract, bloodstream and soft tissue. Clinical specimens were collected in intensive care units, clinical and surgical wards as well as from outpatients as a standard care procedure and were stored for this work. This study was approved by the Ethical Review Board from our institution (Comitê de Ética em Pesquisa – FAMERP) under protocol # 3131/2009.

### Susceptibility testing and phenotypic detection of MBL production

Antimicrobial susceptibility to aztreonam, ceftazidime, cefepime, imipenem, meropenem, piperacillin/tazobactam, amikacin, gentamicin, ciprofloxacin, levofloxacin and polymyxin B were determined by the disk diffusion method, following Clinical and Laboratory Standards Institute recommendations [20]. Phenotypic detection of MBLs was performed by double-disc synergy test (DDST) using the 2-mercaptopropionic acid as a MBL inhibitor [17].

### Genotypic detection of MBL and ESBL genes

Specific primers and protocols were used to detect and sequence *bla*_TEM_*bla*_SHV_*bla*_CTX-M_*bla*_GES_*bla*_KPC_*bla*_IMP_ and *bla*_VIM_[17,21-24]. The detection and sequencing of *bla*_SPM-1_ was performed using primers previously described [25] and also others specifically designed for this study using DS Gene 2.0 Software (Accelrys, USA). PCR products were purified in ethanol according to previously described methodology [26] and subjected to direct sequencing with the ABI PRISM 3130 automated sequencer (Applied Biosystems, Foster City, CA). The products were aligned with Accelrys Gene 2.0 (Accelrys Software Inc. 2006). Database similarity searches were run with BLAST at the National Center for Biotechnology Information website ( http://www.ncbi.nlm.nih.gov).

### Molecular typing

Genetic relatedness was evaluated by pulsed-field gel electrophoresis (PFGE) using SpeI (Fermentas Life Sciences, MD, EUA) with a CHEF-DR II apparatus (Bio-Rad, Richmond, CA, EUA) as described elsewhere [27]. BioNumerics software (Applied Maths) was used for dendrogram construction and clustering, based on the band-based Dice’s similarity coefficient and using the unweighted pair group method (UPGMA) using arithmetic averages. Isolates were considered to belong to the same cluster when similarity coefficient was 90% [28]. The visual Tenover criteria were also applied [29].

## Results

A total of 80.3% (45/56) of isolates were resistant to the combination piperacillin/tazobactam, 62.5% (35/56) to aztreonam, 78.6% (44/56) to ceftazidime, 96.4% (54/56) to cefepime, 96.4% (54/56) to imipenem, 75.0% (42/56) to meropenem, 51.8% (29/56) to amikacin, 82.1% (46/56) to gentamicin, 78.6% (44/56) to ciprofloxacin and 85.7% (48/56) to levofloxacin. All isolates showed sensitivity to polymyxin B (data not shown). Thirteen isolates (23.2%) presented MBL phenotype by the DDST (Table 1).

**Table 1:** Antimicrobial susceptibility of carbapenem resistant P. aeruginosa *

**Isolate**					**Antimicrobials (zone diameter – mm)**									**DDST**	***bla *genotype**
**No**	**Date**	**Unit**	**Infection site**	**CAZ**	**FEP**	**TZP**	**IPM**	**MEM**	**ATM**	**GEN**	**AK**	**CIP**	**LEV**		
				**R ≤ 14**	**R ≤ 14**	**R ≤ 17**	**R ≤ 13**	**R ≤ 13**	**R ≤ 15**	**R ≤ 12**	**R ≤ 14**	**R ≤ 15**	**R ≤ 13**		
				**S ≥ 18**	**S ≥ 18**	**S ≥ 18**	**S ≥ 16**	**S ≥ 16**	**S ≥ 22**	**S ≥ 15**	**S ≥ 17**	**S ≥ 21**	**S ≥ 17**		
Pa01	06/2009	CI	Urinary tract	6	6	6	10	8	10	6	6	6	6	N	*bla*_CTX-M 2_
Pa02	06/2009	G-ICU	Lung	18	6	6	6	6	17	6	6	6	6	N	*bla*_IMP-1_, *bla*_CTX-M 2_
Pa03	06/2009	S-ICU	Urinary tract	20	6	18	6	13	12	6	15	6	6	P	*bla*_IMP-1_
Pa04	06/2009	G-ICU	Lung	6	6	6	12	14	16	6	19	6	6	N	-
Pa05	07/2009	E-ICU	Blood	6	6	19	6	6	22	6	18	6	6	N	*bla*_SPM-1_
Pa06	07/2009	E-ICU	Lung	6	6	10	11	14	14	12	16	6	6	N	-
Pa07	07/2009	N-Dep	Lung	6	6	6	6	6	16	6	6	6	6	N	-
Pa08	07/2009	C-Dep	Urinary tract	6	6	6	6	16	8	6	6	6	6	N	-
Pa10	07/2009	Outpatient	Urinary tract	6	6	13	6	6	23	6	15	6	6	N	*bla*_CTX-M 2_
Pa11	07/2009	G-ICU	Lung	6	6	14	6	6	22	6	18	6	6	P	*bla*_SPM-1_
Pa12	08/2009	E-ICU	Lung	6	6	16	6	6	19	6	21	6	6	P	*bla*_SPM-1_
Pa14	08/2009	C-Dep	Urinary tract	6	6	6	10	15	10	6	18	6	6	N	-
Pa17	08/2009	P-ICU	Lung	17	6	16	13	17	16	6	6	6	6	N	*bla*_CTX-M 2_
Pa18	08/2009	E-ICU	Lung	6	6	6	6	6	12	6	17	6	6	P	*bla*_SPM-1_
Pa19	08/2009	P-ICU	Lung	6	6	6	6	6	14	19	23	23	10	N	-
Pa22	08/2009	SW	Skin	19	6	16	6	18	8	6	6	6	6	N	*bla*_CTX-M 2_
Pa24	08/2009	C-Dep	Urinary tract	6	6	6	6	6	6	6	18	6	6	N	-
Pa27	08/2009	G-ICU	Catheter tip	6	6	10	10	15	8	6	19	6	6	N	-
Pa28	08/2009	G-ICU	Urinary tract	6	6	6	6	6	14	6	13	6	6	P	*bla*_SPM-1_
Pa29	09/2009	C-Dep	Lung	23	6	17	6	22	12	6	6	6	6	N	-
Pa30	09/2009	P-ICU	Eyes	6	6	18	6	6	9	17	23	22	13	N	-
Pa31	09/2009	P-ICU	Abdominal	6	6	6	6	6	19	19	20	15	6	N	-
Pa32	09/2009	C-Dep	Lung	6	6	12	6	19	13	6	6	6	6	N	-
Pa33	09/2009	P-ICU	Lung	6	6	6	6	6	13	19	22	20	6	N	-
Pa34	09/2009	C-ICU	Bone	6	6	6	6	6	12	6	6	6	6	N	-
Pa35	09/2009	C-ICU	Lung	18	6	21	6	6	6	6	12	26	30	N	-
Pa36	09/2009	N-Dep	Lung	6	6	18	6	13	6	6	6	17	25	N	-
Pa37	09/2009	S-ICU	Urinary tract	6	6	14	6	6	20	6	20	6	6	P	*bla*_SPM-1_
Pa39	09/2009	G-ICU	Catheter tip	25	6	14	6	24	21	6	6	6	6	N	-
Pa40	09/2009	P-ICU	Urinary tract	6	6	19	6	6	9	14	20	31	27	N	-
Pa41	09/2009	E-ICU	Lung	20	15	6	6	18	23	6	6	6	6	N	-
Pa42	09/2009	N-ICU	Eye	6	6	25	14	16	23	6	20	30	28	N	-
Pa43	10/2009	S-ICU	Lung	6	6	6	6	14	12	6	18	6	6	N	-
Pa44	10/2009	G-ICU	Lung	17	6	20	10	11	15	16	18	23	18	N	-
Pa45	10/2009	E-ICU	Skin	21	6	15	10	11	10	6	6	6	6	N	-
Pa47	11/2009	SW	Urinary tract	6	15	10	6	6	23	6	23	6	6	P	*bla*_SPM-1_
Pa49	10/2009	N-ICU	Urinary tract	6	6	27	6	6	23	6	6	33	30	P	*bla*_IMP-1_
Pa50	10/2009	SC	Bone	6	6	6	6	12	10	6	15	6	6	N	-
Pa51	10/2009	S-ICU	Urinary tract	6	6	6	6	19	18	6	6	6	6	N	-
Pa53	10/2009	C-UTI	Lung	20	6	18	6	6	14	6	6	6	6	N	*bla*_IMP-1_, *bla*_CTX-M 2_
Pa54	10/2009	E-ICU	Urinary tract	6	6	6	6	6	17	6	16	6	6	P	*bla*_SPM-1_
Pa55	11/2009	ER	Urinary tract	6	6	6	6	6	17	6	6	6	6	N	*bla*_CTX-M 2_
Pa57	11/2009	S-ICU	Urinary tract	26	6	6	6	6	25	6	6	6	6	N	*bla*_CTX-M 2_
Pa58	11/2009	E-ICU	Urinary tract	6	6	6	6	6	21	6	18	6	6	P	*bla*_SPM-1_
Pa59	11/2009	E-ICU	Lung	6	6	22	6	6	21	14	17	20	20	P	*bla*_IMP-1_*, bla*_GES-1_
Pa60	11/2009	ER	Lung	6	6	10	12	6	8	6	6	6	6	N	*bla*_IMP-1_, *bla*_CTX-M 2_
Pa61	11/2009	N-Dep	Blood	6	6	6	6	6	10	6	6	6	6	N	*bla*_CTX-M 2_
Pa62	11/2009	E-ICU	Lung	6	6	6	13	6	11	6	6	6	6	P	*bla*_IMP-1_
Pa63	11/2009	E-ICU	Lung	6	6	15	6	6	6	6	19	6	6	N	-
Pa64	11/2009	N-Dep	Tendon	6	6	6	6	6	12	6	6	6	6	N	*bla*_GES-5_
Pa65	11/2009	S-ICU	Lung	6	6	6	6	6	11	6	6	6	6	N	-
Pa66	11/2009	C-ICU	Lung	6	6	15	6	6	14	13	20	30	25	N	-
Pa67	11/2009	ER	Urinary tract	6	6	6	6	6	15	13	6	6	6	N	-
Pa69	11/2009	E-ICU	Lung	6	6	6	19	6	6	21	6	20	6	P	*bla*_SPM-1_
Pa70	11/2009	E-ICU	Lung	6	6	6	6	11	11	12	6	6	6	N	-
Pa71	11/2009	E-ICU	Lung	6	6	6	6	6	6	6	6	6	6	N	*bla*_CTX-M 2_

*CAZ *, ceftazidime; *FEP *, cefepime; *TZP *,piperacillin-tazobactam; *IMP *, imipenem; *MEM *, meropenem; *ATM *, aztreonam, *GEN *, gentamicin, *AK *, amikacin; *CIP *,ciprofloxacin; *LEV *, levofloxacin; ^*† *^*DDDS *, double disc synergy test to detect MBL production , *N *, negative, *P *, positive, *ER *, emergence room; *E-ICU *, emergence intensive care unit; *G-ICU *, geral intensive care unit; *S-ICU *, semi-intensive care unit; *C-ICU *, cardiology intensive care unit; *P-ICU *, pediatrics intensive care unit, *N-ICU *, neonatal intensive care unit *N-Dep *, neurology department; *C-Dep *, cardiology department; *SC *, surgical ward, *CI *, cancer institute; *R *, resistant; *S *, susceptible.

The prevalence of isolates harboring MBL genes was 30.3% (17/56). Ten (17.8%) presented the *bla*_SPM-1_ gene, and the *bla*_IMP-1_ was detected in seven isolates (12.5%). In all thirteen isolates presenting MBL phenotype by DDST, *bla*_SPM-1_ or *bla*_IMP-1_ was detected. In addition, the *bla*_IMP-1_ gene was also detected in three isolates which did not display any MBL phenotype. These isolates also presented the *bla*_CTX-M-2_ gene (Table 1). No *bla*_VIM_ type was detected.

Genes encoding ESBLs were detected in 23.2% (13/56) of the isolates. The *bla*_CTX-M-2_ was detected in eleven isolates (19.6%), *bla*_GES-1_ was detected in one and *bla*_GES-5_ was detected in one isolate. Co-existence of *bla*_CTX-M-2_ with *bla*_IMP-1_ was detected in three isolates and *bla*_GES-1_ with *bla*_IMP-1_ in one isolate (Table 1). No *bla*_TEM_ and *bla*_SHV_ types were detected GenBank Accession Numbers are provided in Supplemental Material (Additional File 1).

**Figure 1:**
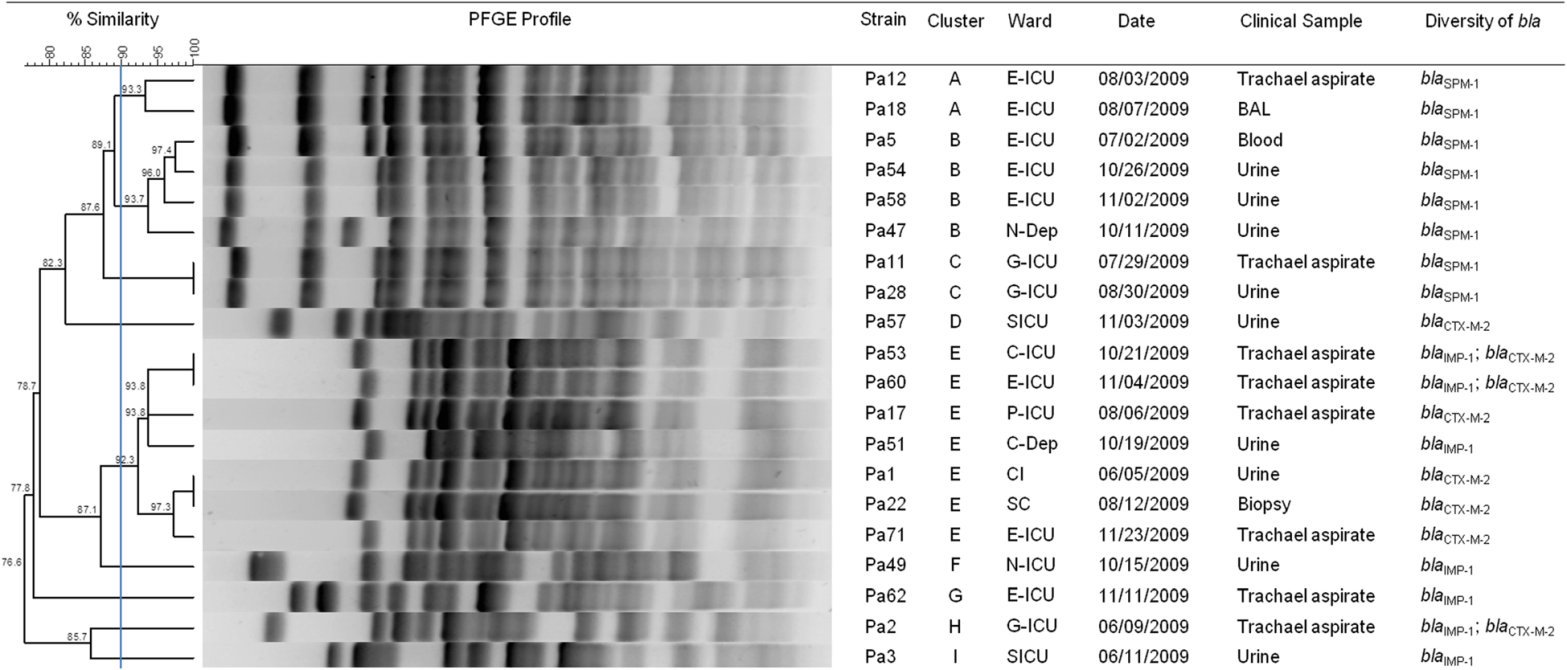
**Dendrogram of PFGE patterns presented by carbapenem resistant *P. aeruginosa *included in this study. ** ER, emergence room; E-ICU, emergence intensive care unit; G-ICU, geral intensive care unit; S-ICU, semi-intensive care unit; C-ICU, cardiology intensive care unit; P-ICU, pediatrics intensive care unit, N-ICU, neonatal intensive care unit N-Dep, neurology department; C-Dep, cardiology department; SC, surgical ward, CI, cancer institute.

The degree of relatedness among the *Pseudomonas aeruginosa* harboring MBL and ESBL genes was assessed by PFGE analysis (Figure 1). Eight isolates harboring *bla*_SPM-1_ were typed, and by visual inspection all were considered closely related, presenting up to three bands of difference. According to PFGE profiles these isolates were grouped within three clusters (A, B and C). Similarity between cluster A and B was of 89.1%. Clusters A and B presented 87.6% of similarity with cluster C.

The *P. aeruginosa* harboring *bla*_IMP-1_ were distributed among five different clusters (E to I), and isolates harboring *bla*_CTX-M-2_ in three clusters (D, E, H). Cluster E included seven isolates presenting similarity of at least 92.3%. Within this cluster, four isolates exhibited *bla*_CTX-M-2_, one exhibited *bla*_IMP-1_ and two isolates exhibited *bla*_IMP-1_ and *bla*_CTX-M-2_ simultaneously. One isolate harboring *bla*_CTX-M-2_ was placed in cluster D, four isolates harboring *bla*_IMP-1_ were placed in clusters F, G, H and I, and one isolate exhibiting *bla*_IMP-1_ and *bla*_CTX-M-2_ simultaneously was placed in cluster H. Isolates harboring *bla*_GES-1_ and *bla*_GES-5_ could not be typed.

## Discussion

The pattern of multidrug resistance presented by the *P. aeruginosa* included in this study was expected, since carbapenem resistant strains exhibit a broad-spectrum resistance to beta-lactams and often present resistant phenotype to additional classes of drugs. Also, acquired ESBL and MBL genes typically group with other drug resistance determinants in the variable region of multi-resistance integrons [30-32]. Furthermore, *P. aeruginosa* isolated in Latin America, including Brazil, are reported as presenting the higher rates of antibiotic resistance [33]. *P. aeruginosa* strains resistant to polymyxins were not detected in this study. However, this result should be confirmed by a dilution method, considering that the disc diffusion technique, commonly used in clinical microbiology laboratories is reported to be an unreliable method for evaluating the susceptibility to polymyxins because these drugs diffuse poorly into agar [34].

In this study MBL genes were detected in 26.7% of isolates, indicating that despite the increasing significance of MBL production among *P. aeruginosa*, this was not the main mechanism of resistance to imipenem and/or meropenem. This reality was previously reported in another Brazilian hospital [35]. In fact, resistance to carbapenems in *P. aeruginosa* is due not only to the production of carbapenemases, but also to different mechanisms such as upregulation of multidrug efflux pumps, cell wall mutations leading to reduction in porin channels and production of different beta-lactamases [1,11]. Expression of these mechanisms, isolated or in combination, may cause variation in rates of resistance to imipenem and meropenem, observed in this study [5]. The twelve isolates presenting MBL phenotype by the DDST harbored MBL genes (*bla*_SPM-1_ or *bla*_IMP-1_), confirming the efficacy of this method for the phenotypic detection of MBL producing strains [17,36].

Diversity of MBL among *P. aeruginosa* varies by regional areas [37]. In this study the 17.5% of the carbapenem resistant isolates presented the *bla*_SPM-1_ gene, and *bla*_IMP-1_ was detected in 10.5% of the isolates. SPM-1 is in fact the most prevalent MBL in Brazil [38]. In a different way, IMP-1 producing strains have been reported in various Brazilian hospitals in diverse rates [35,39,40].

Molecular typing by PFGE showed a wide diversity of patterns. All isolates exhibiting *bla*_SPM-1_, a chromosomally located gene [41,42] presented high coefficient of similarity and closely related PFGE patterns, suggesting a close genetic relationship. This is in agreement with previously described data, showing that the Brazilian SPM-1-producing *P. aeruginosa* are probably derived from a single clone [43]. According to the same study, even a recent genomic variety recently observed among various SPM-1-producing *P.aeruginosa* isolates is result of the accumulation of mutations along the time.

Occurrence of *bla*_IMP-1_ in *P. aeruginosa* described in this study is likely a result of gene horizontal transferring, since isolates harboring *bla*_IMP-1_ belong to five different clusters. Dissemination of *bla*_IMP_ among Gram-negative pathogens is mediated by mobile elements of DNA, explaining why the same gene might be detected in different strains. The genes for IMP enzymes are often carried as cassettes within integrons, which facilitate recombination and facilitate rapid horizontal transferring [44-46].

The coexistence of *bla*_IMP-1_ with *bla*_CTX-M-2_ in *P. aeruginosa* was, at the best of our knowledge, detected for the first time. Interestingly, the three isolates presenting this combination of genes did not provide a positive result in the DDST test, and were the only *bla*_IMP-1_ carriers in which MBL phenotypic detection was unsuccessful. Since ceftazidime is poorly hydrolyzed by CTX-M-2 [31], we could not assume that inactivation of this antimicrobial, used as substrate, may have been responsible for this result. However, simultaneous production of MBLs and ESBLs by the same strain is known to promote further complexity in phenotypic detection [7,47,48]. This result emphasizes the importance of molecular methods for the identification of antimicrobial resistance in multidrug resistant pathogenic bacteria. In this matter, detection of resistance genes by PCR may provide clinically relevant information with positive impact in patient prognosis [18].

The detection of *bla*_CTX-M-2_*bla*_GES-1_ and *bla*_GES-5_ in this study reflects the recent emergence of ESBLs in *P. aeruginosa* as result of the great dissemination capability exhibited by these genes that occur mostly as part of integron structures on mobile transmissible genetic elements [49-52]. Location of *bla*_CTX-M-2_ in *P. aeruginosa* is believed to be a result of their transfer from *Enterobacteriaceae*[51]. Recently, a high prevalence of *K. pneumoniae* harboring *bla*_CTX-M-2_ in the same hospital was reported [21], and this may have been the reservoir for horizontal transmission.

Regarding GES-type ESBLs, although these enzymes are not considered as primary β-lactamases in *P. aeruginosa*, acquisition in conditions of antimicrobial pressure may be beneficial [52]. The *bla*_GES-1_ was detected in co-existence with *bla*_IMP-1_, and *bla*_GES-5_ was detected in a strain resistant to imipenem and meropenem, but presenting no MBL phenotype. Considering that GES-5 presents enhanced hydrolyse activity against carbapenems [10], it is likely that GES-5 production contributed for the carbapenem resistance in this isolate.

## Conclusion

Production of MBLs was not the main mechanism of resistance to carbapenems among the studied strains. However, IMP-1 is disseminated among different strains of carbapenem resistant *P. aeruginosa*, and intra-hospital spread of SPM-1 producing strains was observed. The detection of *bla*_CTX-M-2_, *bla*_GES-1_ and *bla*_GES-5_ and the unique coexistence of *bla*_CTX-M-2_ with *bla*_IMP-1_ and *bla*_GES-1_ with *bla*_IMP-1_ exemplify the extraordinary ability presented by *P. aeruginosa* to acquire multiple resistance mechanisms.

Clonal dissemination of multidrug resistant *P. aeruginosa* within the hospital was inferred by the observation of strains presenting the identical PFGE profiles infecting different patients, and the occurrence of clonal dissemination in different ICUs and wards. This finding enhances the necessity of better observance of infection control practices, to prevent further dissemination of this challenging pathogen.

## Competing interests

The authors declare that they have no competing interests.

## Authors’ contribution

MP, TC and MCLN carried out the molecular genetic studies, participated in the sequence alignment. MGL, FGR, MTGA, MLN and MCLN participated in the design of the study. MGL, MTGA and MP collected and characterize the isolates. MLN, MTGA, MCLN and FGR analyzed the results. MP, MLN and MCLN drafted the manuscript. All authors read and approved the final manuscript.

## Pre-publication history

The pre-publication history for this paper can be accessed here:

http://www.biomedcentral.com/1471-2334/12/176/prepub

## Supplementary Material

Additional file 1 *Gen Bank Accession Number – Sup Material.* Click here for file
