# Supplementary material for: Detection of P. aeruginosa harboring bla CTX-M-2, bla GES-1 and bla GES-5, bla IMP-1 and bla SPM-1 causing infections in Brazilian tertiary-care hospital
Source: BMC Infect Dis. 2012 Aug 3;12:176. doi: 10.1186/1471-2334-12-176 (PMC3512492; doi:10.1186/1471-2334-12-176)
Supplement: Additional file 1 — Gen Bank Accession Number – Sup Material. [file 1471-2334-12-176-S1.doc]

*Gen Bank Accession Number – Sup Material*

GU929907: PHB 1 beta-lactamase CTX-M-2

GU831546: PHB 2 metallo-beta-lactamase IMP-1

GU929908 PHB 2 beta-lactamase CTX-M-2

GU831547: PHB 3 metallo-beta-lactamase IMP-1

GU831554 PHB 5 metallo-beta-lactamase SPM-1

GU831555 PHB 11 metallo-beta-lactamase SPM-1

GU831556 PHB 12 metallo-beta-lactamase SPM-1

GU831557 PHB 18 metallo-beta-lactamase SPM-1

GU929910 PHB 17 beta-lactamase CTX-M-2

GU929911 PHB 22 beta-lactamase CTX-M-2

GU831558 PHB 28 metallo-beta-lactamase SPM-1

GU831560 PHB 47 metallo-beta-lactamase SPM-1

GU831548: PHB 49 metallo-beta-lactamase IMP-1

GU831549: PHB 51 metallo-beta-lactamase IMP-1

GU929917: PHB 53 beta-lactamase CTX-M-2

GU831550: PHB 53 metallo-beta-lactamase IMP-1

GU831561: PHB 54 metallo-beta-lactamase SPM-1

GU831562: PHB 58 metallo-beta-lactamase SPM-1

GU831551: PHB 59 metallo-beta-lactamase blaIMP-1

GU831563: PHB 59 beta-lactamase blaGES-1

GU929913: PHB 57 beta-lactamase CTX-M-2

GU929914: PHB 60 beta-lactamase CTX-M-2

GU831552: PHB 60 metallo-beta-lactamase IMP-1

GU929915 PHB 61 beta-lactamase CTX-M-2

GU831553 PHB 62 metallo-beta-lactamase IMP-1

GU831564 PHB 64 beta-lactamase GES-5

GU929916 PHB 71 beta-lactamase CTX-M-2
